# Advances in Gelatin-Based Tissue Engineering Using HRP/H_2_O_2_

**DOI:** 10.3390/gels11060460

**Published:** 2025-06-16

**Authors:** Marino Basha, Ahmad Aburub, Filippos F. Karageorgos, Georgios Tsoulfas, Aleck H. Alexopoulos

**Affiliations:** 1 Department of Transplantation Surgery, Center for Research and Innovation in Solid Organ Transplantation, School of Medicine, Aristotle University of Thessaloniki, 54642 Thessaloniki, Greece; marinompasa@auth.gr (M.B.); aaaburub@auth.gr (A.A.); filipposk@auth.gr (F.F.K.); 2 Chemical Process & Energy Resources Institute, 6th Km Harilaou-Thermi Rd., P.O. Box 60361, 57001 Thessaloniki, Greece

**Keywords:** gelatin, tissue engineering, 3D printing, hydrogel, horseradish peroxidase, hydrogen peroxide, nanofibers, interpenetrating network, microgel, wound healing

## Abstract

Gelatin, a biocompatible and biodegradable polymer, has garnered considerable attention in tissue engineering (TE) due to its diverse applications enabled by its tunable physical properties. Among the various strategies employed for the fabrication of gelatin-based hydrogels, the use of horseradish peroxidase (HRP) and hydrogen peroxide (H_2_O_2_) as a catalytic system has been highlighted as an effective tool for producing hydrogels with highly modifiable properties. Herein, we explore recent progress in the utilization of the HRP/H_2_O_2_ catalytic system for the creation of gelatin-based hydrogels, with an emphasis on TE applications. Particular attention has been given to the interplay between variations in the concentration equilibrium of HRP and H_2_O_2_ and the fine-tuning of gel properties tailored for various TE applications. Emerging trends, such as in situ gelation and hybrid bioinks, have also been examined through the lens of their prospective applications, extrapolating from the findings in cell cultures and animal models. A comprehensive review of two databases (Scopus and Web of Science) was conducted. The data extracted from each study included the materials used for each application, methods used for material preparation, cells used in the TE application, laboratory animals used, and whether computational/simulation techniques were implemented. The applications included both homopolymeric hydrogels, using only gelatin as the backbone, and copolymeric hydrogels, with ≥2 polymers.

## 1. Introduction

Tissue engineering (TE) is an interdisciplinary branch of science that aims to develop innovative strategies for the repair or replacement of damaged tissues and organs [1,2,3], drawing upon the principles of medicine, biology, pharmacy, engineering, materials science, and chemistry [4]. Central to this endeavor is the development of hydrogels that act as three-dimensional scaffolds to support cell proliferation and tissue regeneration. Their utility in TE is highlighted by their highly tunable properties and biocompatibility, which renders them structurally similar to the extracellular matrix. These materials form 3D networks through the crosslinking of polymers, either by chemical means or through physical interactions like chain entanglement and hydrophobic forces. Their ability to absorb large quantities of water, coupled with tunable mechanical properties and controlled degradation rates, makes them ideal candidates for applications in drug delivery, wound healing, and regenerative medicine.

**Figure 1:**
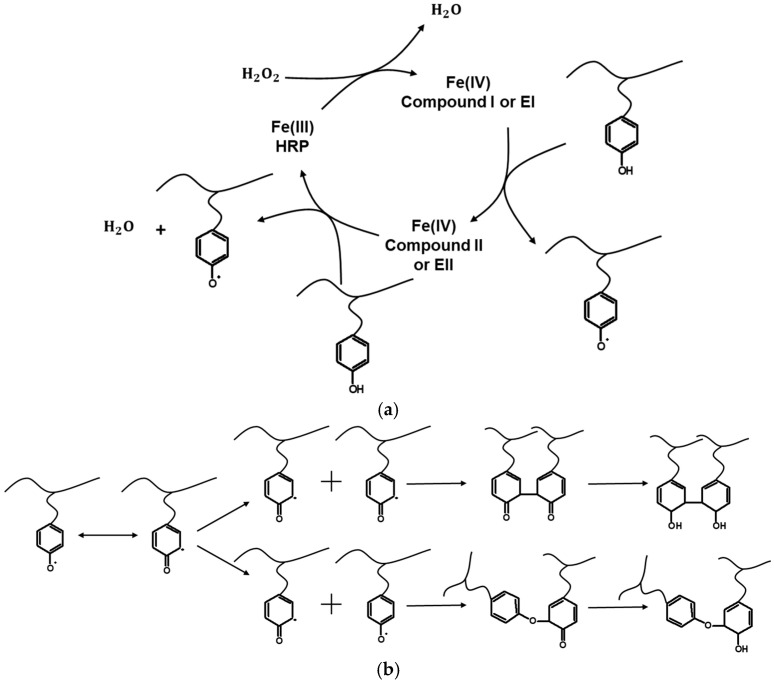
(**a**) Activation of polymer–phenol (e.g., gelatin-tyr or hyaluronic acid-tyr) conjugates in the presence of HRP enzyme and hydrogen peroxide. (**b**) Isomerization of phenolic radicals and crosslinking reactions between different types of “live” polymer chains and enolization of crosslinked polymer chains. The figure was taken from reference [12].

A key concern regarding hydrogel synthesis is the cytotoxicity associated with residual chemical crosslinkers, which limits their utility in biomedical applications. To address this, enzymatic crosslinking methods have been widely adopted, with the HRP/H_2_O_2_ system being among the most prominent, as it minimizes toxic byproducts while enabling precise control over the hydrogel properties. HRP, an enzyme derived from the roots of *Armoracia rusticana*, contains a heme group composed of protoporphyrin and Fe(III), enabling catalytic polymerization, resulting in hydrogel formation. Its applications in biomedical engineering have been studied extensively [5,6,7,8,9,10,11], underscoring its efficacy with monomers such as dextran [6], chitin [7], hyaluronic acid [8], gelatin [9], and chitosan [5]. In particular, the HRP/H_2_O_2_ system has shown great versatility and has been applied both experimentally and through predictive simulations to tailor hydrogel properties for specific tissue-engineering applications. This catalytic system facilitates the formation of covalent bonds between phenol-modified polymer chains, such as tyramine, resulting in biocompatible hydrogels with controlled viscoelasticity, porosity, and degradation rates. Note also that the HRP/H_2_O_2_ system has been studied both experimentally and through predictive simulations for the production of hydrogels [12,13]. The HRP catalytic cycle and hydrogel formation reactions are shown in Figure 1. More information on HRP and its applications can be found in the literature [14,15,16].

Among the many biopolymers explored, gelatin is an especially promising material for TE. Gelatin, derived from the hydrolysis of collagen [17], comprises 18 amino acids, with glycine, proline, and hydroxyproline constituting approximately 57% of its composition. The remainder includes amino acids such as glutamic acid, alanine, arginine, and aspartic acid [18,19]. Its molecular structure includes hydrophilic unfolded chains and a high content of carbon (50.5%), oxygen (25.2%), nitrogen (17%), and hydrogen (6.8%) [19,20]. These characteristics contribute to the excellent biocompatibility, biodegradability, and capacity of gelatin to mimic the natural ECM, making it particularly suitable for scaffolding applications. Owing to these ECM-like properties, gelatin integrates effectively into hydrogel systems, which serve as synthetic ECM analogs by providing structural support and biochemical cues that promote cell attachment and mediate the transmission of mechanical signals essential for regulating cellular behaviors, such as proliferation, migration, differentiation, and spreading. The selection of the monomer, along with the synthetic process, including polymer and crosslinker concentrations, pH, and temperature, is pivotal to the hydrogel’s swelling behavior, degradation kinetics, mechanical strength, and network density.

This review emphasizes recent advancements in HRP/H_2_O_2_-mediated fabrication of gelatin-based hydrogels for tissue engineering purposes [21]. By harnessing the synergistic properties of gelatin and the enzymatic precision of the HRP/H_2_O_2_ crosslinking method, researchers have developed hydrogels with enhanced performance tailored for a variety of biomedical applications.

## 2. Methodology

### 2.1. Study Design and Search Strategy

Only research papers investigating gelatin-based applications in TE were included, while review articles and book chapters were excluded. No non-English works were included, and no search filter restrictions were applied, such as the region of authors or publication date.

Two databases (i.e., Web of Science (Clarivate (Philadelphia, PA, USA, London, UK))) and Scopus (Elsevier (Amsterdam, The Netherlands)) were used for the search of results. The search terms “TITLE-ABS-KEY (gelatin AND hrp AND h2o2 AND tissue AND engineering)” were used in Scopus. The search terms “gelatin AND hrp AND h2o2 AND tissue AND engineering (Title) OR gelatin AND hrp AND h2o2 AND tissue AND engineering (Abstract) OR gelatin AND hrp AND h2o2 AND tissue AND engineering (Author Keywords)” were used in Web of Science. FFK conducted the literature search, and MB, AA, and FFK selected the articles.

### 2.2. Study Selection and Data Extraction

**Figure 2:**
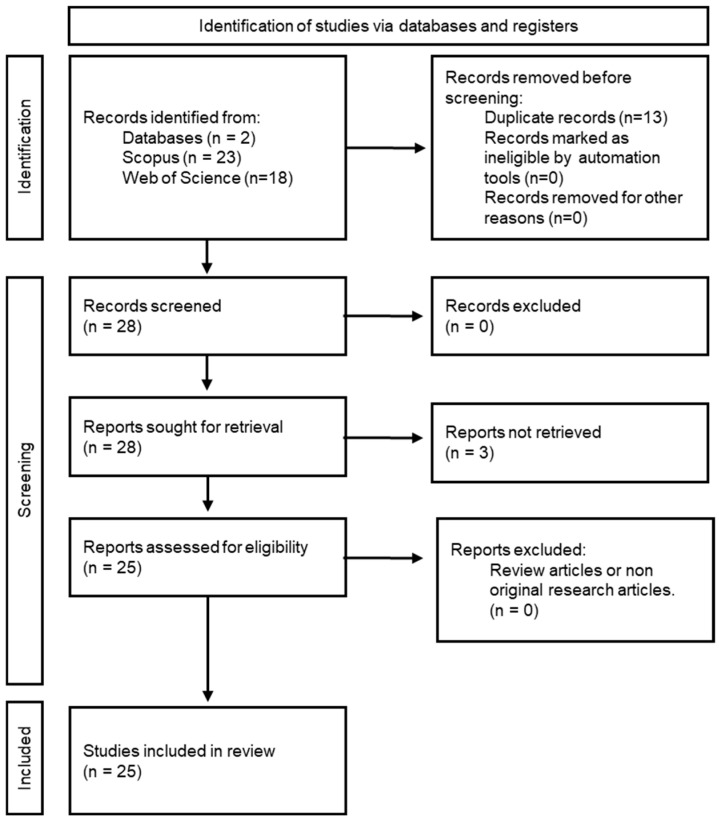
PRISMA flowchart of article selection.

A total of 41 articles were identified. No book chapters or books were found. Of these, 13 articles were duplicates, leaving 28 articles eligible for the study. Three articles were not retrieved; therefore, full-text examination and data extraction were performed in 25 articles [22,23,24,25,26,27,28,29,30,31,32,33,34,35,36,37,38,39,40,41,42,43,44,45,46]. Finally, the PRISMA flowchart [47] leading to the 25 studies considered for this review is shown in Figure 2.

MB, AA, and FFK extracted the relevant data, including the authors, type of study, materials used, cells used, animal models used (if any), tissue engineering application, use of simulation tools or mathematical models, method used for the fabrication of the hydrogel, and a basic outcome summary.

### 2.3. Quality Assessment

Although no formal quality assessment tool was implemented due to the methodological variability of the examined studies, an internal validation process was implemented to ensure data accuracy and reliability. After each author extracted the key information, FFK cross-checked all data against their original sources to maintain accuracy. Subsequently, MB and AA assessed and verified the original draft to ensure its completeness and consistency.

## 3. Applications of HRP/H_2_O_2_-Catalyzed Hydrogels in TE

Hydrogels formed through the HRP/H_2_O_2_ catalytic system are a versatile tool for numerous tissue engineering applications due to their tunable properties, biocompatibility, and similarity to the extracellular matrix found in living tissues. The fabrication process for these hydrogels varies according to the desired properties of the material. The following sections detail the diverse applications of these hydrogels, categorized by the specific tissue or organ targeted for regeneration or repair, along with the relevant synthetic techniques. Table 1 summarizes the useful data extracted from each study.

### 3.1. Wound Healing and Soft Tissue Repair

**Figure 3:**
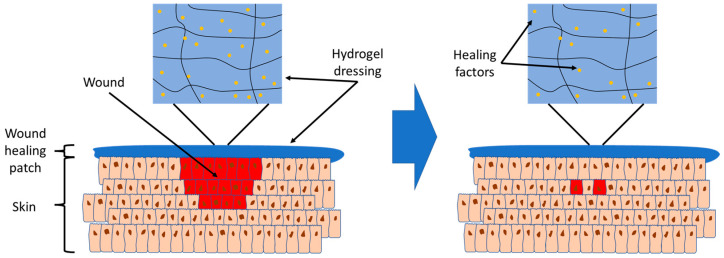
Schematic representation of wound healing using a hydrogel dressing.

HRP/H_2_O_2_ crosslinked hydrogels have shown significant promise in promoting wound healing and facilitating soft tissue regeneration [22,25,34,36,39,43,46]. A gelatin-based hydrogel containing human dermal fibroblasts catalyzed by HRP/H_2_O_2_ presents a novel approach for wound treatment [34]. The hydrogel was prepared using a dual-syringe system that allowed in situ enzymatic gelation directly at the wound site. This method enabled the prolonged survival of fibroblasts and prevented their dispersal from the injection site, which in turn promoted a faster closure of wounds with higher collagen deposition and neovascularization [34]. Gelatin-hydroxyphenylpropionic acid (GH) was used as the backbone of the hydrogel, and varying concentrations of HRP and H_2_O_2_ were tested to evaluate the effect of stiffness on cellular behavior [34]. Soft hydrogels (1.1 kPa) yielded substantially better results than their stiffer counterparts (6.6 kPa), with optimal concentrations of HRP = 0.02 mg/mL and H_2_O_2_ = 0.007 wt%. Specifically, soft hydrogels offer nearly double the moisture retention and support enhanced fibroblast proliferation, spreading, and network formation without impeding the production of extracellular matrix, unlike stiffer hydrogels [34]. Figure 3 shows a schematic representation of the wound-healing process.

Similarly, a dual-network hydrogel composed of gelatin derived from tilapia skin and fucoidan reinforced with agarose has shown promising potential for wound dressing applications, as it possesses favorable capabilities, being highly biocompatible and stable while also functioning as a topical antioxidant [22]. For its synthesis, tyramine was first conjugated to tilapia skin gelatin and fucoidan, with the phenolic groups of the derivatives forming covalent bonds catalyzed by the HRP/H_2_O_2_ system [22]. Simultaneously, an agarose solution was cooled to form a secondary hydrogen-bonded network, resulting in a dual network structure [22]. In vivo experiments have demonstrated the ability of this hydrogel to accelerate wound healing, reduce microbial colonization, reduce inflammation, and promote tissue regeneration, highlighting its potential as an alternative to traditional wound dressings [22].

An interpenetrating polymer network (IPN) hydrogel has also been created by simultaneously applying two enzymatic crosslinking reactions, with the aim of enhancing the mechanical strength of hydrogels used in wound dressings [36,38]. This involves combining gelatin and chitosan functionalized with phloretic acid (chitosan-PA) with transglutaminase (TG) and HRP in the presence of a low concentration of H_2_O_2_ [36]. TG catalyzes the formation of amide bonds between glutamine and lysine residues on adjacent gelatin chains, while HRP catalyzes the crosslinking of phenol groups in chitosan-PA [36]. This dual-network approach resulted in enhanced mechanical properties compared to gelatin hydrogels crosslinked by TG alone, without affecting the functionality of the hydrogel, as indicated by the non-inferior adhesion and proliferation of L929 cells [36,38]. Fibers of these bi-enzymatically crosslinked IPN structures have notably high tensile strength and elasticity, which is directly related to the gelatin and chitosan-PA content, with a significant feature being their knittability under wet spinning conditions [38].

Further highlighting the prospective value of HRP-crosslinked systems in wound healing, gelatin–poly(ethylene glycol)–tyramine (GPT) hydrogels were developed with an embedded angiogenic peptide (SVVYGLR) to enhance neovascularization and soft-tissue regeneration [43]. These hydrogels were formed via HRP/H_2_O_2_ crosslinking and demonstrated tunable physical properties, which were dependent on both the H_2_O_2_ concentration and the amount of peptide loaded within them [43]. The presence of SVVYGLR significantly boosted the in vitro activity of human umbilical vein endothelial cells (HUVECs), and in vivo studies confirmed an increase in angiogenesis at the sites of hydrogel injection [43]. As blood vessel formation is critical for effective wound healing, this modality has potential in the field of regenerative medicine [43].

In addition to wound treatment, hydrogels have been successfully used in hemostatic applications. In particular, a catechol-based bioadhesive was developed using amine-rich, highly branched gelatin functionalized with catechol groups via a reaction with 3,4-dihydroxyphenylacetic acid [24]. The end-product catechol bioadhesive was formed via two types of crosslinking: HRP/H_2_O_2_-catalyzed covalent bonds and catechol-Fe^3+^ coordinate bonds [24]. In vivo testing in mice confirmed its efficacy in stopping hemorrhage from severed carotid arteries, liver incisions, and penetrating heart wounds while promoting the healing of these vital organs [24]. Likewise, a dopamine-modified methacrylate gelatin (GMDA) hydrogel, synthesized through a two-step crosslinking process, could be used in minimally invasive procedures because of its hemostatic properties and ability to prevent wound infections [39]. Its fabrication begins with the synthesis of methacrylated gelatin (GelMA), followed by the transfer of dopamine onto GelMA to create GMDA [39]. Gelation of GMDA was achieved by initial crosslinking with HRP/H_2_O_2_, followed by UV light exposure (365 nm) to induce photo-crosslinking and form the final hydrogel [39]. This unique hydrogel has strong adhesive properties and provides antibacterial protection due to residual H_2_O_2_ while being injectable, a feature that allows it to conform to irregular wounds [39]. Its hemostatic performance was notable, as it could withstand blood pressures up to 250 mmHg [39].

Finally, a bioactive wound dressing with advanced antimicrobial and healing capabilities was developed using gelatin-grafted dopamine (GT-DA) and polydopamine-coated carbon nanotubes (CNT-PDA) in conjunction with doxycycline [46]. GT-DA and chitosan were mixed with CNT-PDA solutions, and HRP catalyzed the polymerization in the presence of H_2_O_2_ [46]. This hydrogel exhibited powerful antimicrobial capabilities due to the presence of doxycycline and the photothermal effect of CNT. Additionally, it has enhanced adhesive properties attributed to its dopamine content and antioxidant action, showing favorable in vitro and in vivo behaviors [46].

### 3.2. Bone and Cartilage TE

The tunable properties of HRP/H_2_O_2_ crosslinked hydrogels make them particularly suitable for bone and cartilage regeneration, where mechanical parameters and scaffold properties are critical for cell differentiation and tissue formation. A biomimetic hydrogel was developed using chondroitin sulfate tyramine and tyramine-modified gelatin enzymatically crosslinked with HRP and H_2_O_2_ [23]. Specifically, tyramine-functionalized chondroitin sulfate (CDTA) and tyramine-functionalized gelatin (GTA) were produced separately [23]. The GTA-CDTA hybrid hydrogels were formed in situ by mixing solutions containing GTA, CDTA, and HRP with solutions containing GTA, CDTA, and H_2_O_2_ [23]. The incorporation of biphasic calcium phosphate (BCP) nanoparticles into this hydrogel enables its use as an injectable system with enhanced biomineralization properties and a porous structure conducive to bone growth [23]. In vitro studies have demonstrated the non-toxicity of the hydrogel and its potential to enhance calcium deposition and osteogenic activity in mesenchymal cells, making it a promising material for bone repair and osteochondral tissue engineering [23].

Hydrogel stiffness is a major determinant of its efficacy in promoting bone and cartilage regeneration [27,28,30,31]. An injectable hydrogel scaffold system with tunable stiffness, composed of a gelatin-hydroxyphenylpropionic acid conjugate, was formed using the oxidative coupling of hydroxyphenylpropionic acid moieties catalyzed by H_2_O_2_ and HRP [31]. The conjugate was synthesized using a carbodiimide/active ester-mediated coupling reaction [31]. The stiffness of these hydrogels was precisely tuned by adjusting the H_2_O_2_ concentration, which in turn affected human mesenchymal stem cell behavior [30,31]. Chondrocytes grown in medium-stiffness hydrogels showed the highest levels of sulfated glycosaminoglycan production and an improved collagen II to collagen I gene expression ratio, which translated into optimal tissue repair and hyaline cartilage formation in medium-stiffness hydrogels compared to low- and high-stiffness hydrogels when tested in rabbit models [28]. The utilization of a gelatin-hydroxyphenylpropionic acid-tyramine conjugate instead of gelatin-hydroxyphenylpropionic acid has been shown to expand the limited range of hydrogel stiffness associated with the latter [30]. Specifically, this modification broadened the storage modulus range from 1000–13,500 Pa for Gelatin-HPA hydrogels to 600–26,800 Pa, with the gain in stiffness leading to the upregulation of osteocalcin and runt-related transcription factor 2 expression, which are key indicators of osteogenic differentiation in human mesenchymal stem cells [30]. The production of gelatin-hydroxyphenylpropionic acid-tyramine hydrogels starts with the synthesis of the gelatin-hydroxyphenylpropionic acid conjugate, followed by further conjugation with tyramine [30]. Hydrogel formation is then catalyzed by the HRP/H_2_O_2_ system [30]. Beyond mechanical tuning, the functionality of gelatin-hydroxyphenylpropionic acid hydrogels can be enhanced by conjugating signaling molecules that promote desired biological outcomes. Platelet-Derived Growth Factor-BB and Stromal Cell-Derived Factor 1-α have been encapsulated in polyelectrolyte complex nanoparticles and incorporated into a gelatin-hydroxyphenylpropionic acid matrix prior to crosslinking [27]. This approach facilitated the migration, proliferation, and osteogenic differentiation of bone marrow-derived mesenchymal stem cells, highlighting the potential of this system for enhanced bone regeneration [27].

**Figure 4:**
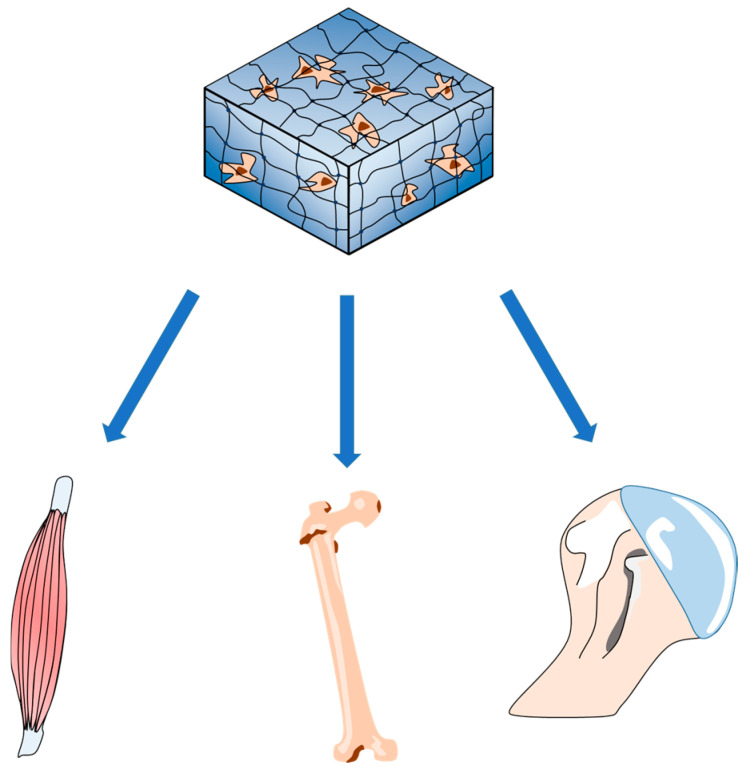
Schematic representation of gelatin-based applications in muscle, bone, and cartilage TE. Figure adapted from [48].

Hybrid hyaluronic acid/gelatin microgels have been explored as a potential substitute for autologous bone grafts [40]. These microgels were created by first conjugating tyramine to hyaluronic acid and gelatin and then crosslinking through HRP/H_2_O_2_, with the concentrations of the reactants optimized for a 10-s gelation time [40]. The formation of spherical microglobules was achieved using a “water-in-oil technique”, with a surfactant to facilitate rapid droplet formation [40]. The size of the spheres was inversely related to the stirring speed, with 80–100 µm being the appropriate size for cell delivery [40]. These microgels can be injected through needles and support the differentiation of MG-63 cells, indicating their potential as injectable micro-scaffolds in bone tissue engineering [40]. Figure 4 shows a schematic representation of gelatin-based bone and cartilage TE applications.

### 3.3. Muscle Regeneration and Nerve Repair

The utilization of hydrogels is expanding into more areas of TE, with HRP/H_2_O_2_ mediated ones showing promise in more specialized applications, such as muscle and nerve regeneration. The impact of H_2_O_2_ exposure duration during the fabrication of gelatin–phenol hydrogels is of particular interest, as it has been proven to be a key component of stiffness modulation, with significant implications in the behavior of myoblasts (C2C12 cells), human adipose-derived stem cells (hADS cells), and rat fibroblast 3Y1 cell cultures [26,35]. An aqueous solution containing 3.0% *w*/*v* Gelatin-Ph and 1 U/mL HRP in PBS was added to a polydimethylsiloxane (PDMS) mold [35]. Air containing H_2_O_2_ was then exposed for 15, 30, 45, and 60 min, resulting in hydrogel formation [35]. Stiffness peaked at 30 min of H_2_O_2_ exposure, with further exposure leading to its reduction, either through HRP inactivation by excess H_2_O_2_ or H_2_O_2_-induced degradation of the gelatin. Hydrogels with optimal stiffness, produced through controlled H_2_O_2_ exposure, significantly enhanced myoblast differentiation into myotubes, underscoring the importance of mechanical tuning in muscle tissue engineering [26,35]. These findings highlight the potential of fine-tuning crosslinking conditions to tailor hydrogel properties for improved cell adhesion, viability, and differentiation [26,35].

A novel technique with implications for the treatment of peripheral nerve damage proposes the alignment of severed nerve endings via ultrasound standing waves, followed by their encapsulation within a rapidly solidifying hydrogel [42]. This hydrogel, composed of gelatin-hydroxyphenylpropionic acid, was enzymatically crosslinked using HRP and H_2_O_2_, with PC12 cells added to the gel precursor prior to solidification [42]. Appropriate ingredient concentrations were selected to ensure that gelation was completed in approximately 90 s, allowing researchers to quickly use ultrasound standing waves to align PC12 cells before gelation occurred [42]. This approach utilizes a hydrogel as a supportive “cast” to maintain nerve-ending alignment, guiding proper reconnection and regeneration, thus offering a minimally invasive route for functional recovery after peripheral nerve injury [42].

### 3.4. Vascular Tissue Engineering

Replicating the complex architecture of hollow organs, such as blood vessels, the trachea, and the ureters, has been a major frontier in TE; however, recent advances in hydrogel fabrication methods have begun to make meaningful progress in addressing this challenge [29,44]. Among the emerging strategies, HRP/H_2_O_2_ crosslinked hydrogels offer significant advantages due to their mild gelation conditions, tunable mechanical properties, and biocompatibility [29,44]. A novel approach was developed to fabricate multilayered tubular hydrogels using a composite of gelatin–tyramine (GT) and silk fibroin (SF), which leverages the thermosensitive behavior of gelatin and the mechanical robustness of SF. SF and GT are dissolved at 50 °C and are then cooled to 35 °C before the addition of HRP. The mixture undergoes thermal gelation within a syringe, followed by enzymatic crosslinking mediated by hydrogen in the HRP/H_2_O_2_ initiation system. For standard hydrogels, exposure to H_2_O_2_ lasts 1 h, after which methanol treatment induces conformational changes in SF, yielding structurally stable enzymatically and methanol-treated SF/GT hydrogels [44]. In contrast, for the fabrication of tubular structures, the composite was exposed to H_2_O_2_ for only 10 min to form a crosslinked outer shell. The construct is then immersed in water at 40 °C, melting the gelatin-rich inner core and creating a hollow lumen. Methanol soaking subsequently stabilizes the structure and induces a bilayered wall with dense outer and porous inner layers [44]. A notable feature of this method is the ability to invert the architecture of the tubular wall layers by introducing methanol into the lumen, thereby offering control over the layer orientation [44].

As a topic of great clinical significance, other methods for creating multilayered vascular tissues have also been proposed with encouraging results. One such method produces alginate-based hydrogel fibers loaded with human endothelial cells, while its surface provides conditions that promote the adhesion of human aortic smooth muscle cells, forming a bilayer structure resembling vascular tissues [29]. The fibers are formed by extruding a solution containing alginate with phenolic hydroxyl moieties, horseradish peroxidase, and endothelial cells into a flow of hydrogen peroxide and gelatin with phenolic hydroxyl moieties, simultaneously crosslinking the alginate to form the fiber and immobilizing gelatin on its surface. The adjustment of the flow rate and HRP concentration enabled precise control of the fiber diameter and gelatin-coating density. Endothelial cell viability within the fibers was high (87.1%), and the presence of gelatin allowed smooth muscle cell adhesion and proliferation on the outer layer. The ability to lyse the fiber on demand using alginate lyase permits the release of intact tubular cell constructs, making this method a promising tool for engineering vascular tissues with an endothelial inner layer and an outer layer of smooth muscle cells.

### 3.5. Gene Delivery and Genome Editing

HRP-catalyzed hydrogels present a novel pathway for gene delivery and genome editing, addressing the limitations of conventional viral and non-viral vectors, including limited nucleic acid packaging due to size restrictions, triggering of immune response, rapid degradation, and non-specific distribution [32]. A promising technique utilizes the electrospinning of gelatin containing phenolic hydroxyl moieties to form nanofibers, which are then insolubilized in the presence of HRP and a low concentration of hydrogen peroxide (16 ppm) [32]. Enzymatic crosslinking rendered the nanofibers insoluble within 30 min, avoiding the cytotoxic effects commonly associated with chemical crosslinkers. Immersing the nanofibers in a Lipofectamine/pDNA complex solution immobilizes the complexes onto the scaffold, providing gene-editing capabilities. Human embryonic kidney-derived HEK293 cells cultured on the resulting scaffolds successfully expressed genome-editing molecules, including Cas9 protein and guide RNA (gRNA), resulting in targeted gene “knock-in” and “knock-out” [32]. The similarity of the scaffolds to the natural extracellular matrix promotes cell attachment and proliferation, as indicated by the higher cell densities observed in scaffold cultures than in culture dishes [32]. Additionally, the ability to deliver multiple pDNA molecules simultaneously permits the targeting of multiple gene loci [32]. Collectively, these characteristics render hydrogels versatile tools for advanced gene therapy applications.

### 3.6. Reducing Immunogenicity for In Vivo Applications

A critical consideration in the use of HRP in TE is its potential to induce an immunogenic response. To address this, an innovative technique was proposed in which HRP was immobilized in a syringe and covalently bonded to porous silica particles (diameter = 70–140 µm) via a polyethylene glycol molecule [41]. These particles remained within the syringe while H_2_O_2_ and the hydrogel precursor flowed through, allowing crosslinking to occur without the enzyme becoming embedded in the hydrogel [41]. This HRP-free hydrogel induced a smaller reaction from activated mouse macrophages in vitro and significantly less inflammation in vivo than HRP-containing hydrogels [41]. Particular attention was paid to matching the stiffness between HRP-free and HRP-containing hydrogels for the experiments, as this parameter is important for in vitro macrophage activation and in vivo tissue response [41]. This innovation has the potential to reduce the immunogenic reactions triggered by this plant-derived enzyme, making HRP-catalyzed hydrogels more suitable for in vivo applications [41].

### 3.7. Advances in Fabrication Techniques and Biomaterials

The precise control offered by HRP/H_2_O_2_-mediated crosslinking makes hydrogels produced using this technique valuable for bioprinting complex tissue structures. Nevertheless, fidelity to the prearranged structural parameters is compromised by the effect of gravity on the fluid hydrogel precursors [33]. A novel HRP-mediated extrusion bioprinting method was developed to improve the printing of low-viscosity bioinks (containing cells, HRP, and phenolated polymers) by intermittently depositing an H_2_O_2_-containing support material [33]. This approach effectively counteracts gravitational deformation during printing, ensuring high structural fidelity and enabling in situ gelation of bioink through enzymatic crosslinking. High rates of cell survival were achieved, with 96% of mouse fibroblasts (10T1/2) surviving the extrusion process and 91% enduring the presence of H_2_O_2_, while human hepatoblastoma cells (HepG2) maintained their morphology and viability for two weeks post-printing [33]. This technique could also be applied to produce scaffolds for cell cultures, but would necessitate the use of hybrid bioinks containing both HA-Ph and gelatin-Ph, as the latter ensures better cell adhesion. When printing is completed, the supporting material can be removed without harming the cells [33].

A common thread across tissue engineering applications is the ability to fine-tune the properties of the hydrogels. In response to this need, a novel technique for hydrogel fabrication has been proposed, starting with the electrospinning of gelatin–hydroxyphenyl propionic acid to produce nanofibers, followed by their insolubilization through enzymatic crosslinking mediated by HRP/H_2_O_2_ [37]. The catalytic reaction occurred in an ethanol-water mixture (volume ratio of 85:15) to prevent the dissolution of the nanofibers [37]. A key benefit of this method compared to chemical crosslinking agents is the lack of cytotoxicity and the ability to fine-tune hydrogel properties through the manipulation of H_2_O_2_ concentrations [37]. The produced scaffolds also showed high porosity, enhanced water-absorbing capacity, complete biodegradability within 4 weeks, and high elasticity, supporting the proliferation and viability of human umbilical vein endothelial cells (HUVECs) [37]. Furthermore, in vivo implantation did not cause notable inflammation and exhibited positive behavior, as it enhanced neovascularization [37].

Advancements in existing biomaterials used for hydrogel fabrication with the HRP/H_2_O_2_ system have been instrumental in improving the mechanical properties and biocompatibility. A key example of such improvements is the crosslinking of silk fibroin (SF) with either SF-TA or gelatin-TA (G-TA), leading to dramatically accelerated gelation kinetics and enhanced mechanical strength compared to pure SF hydrogels, which gel slowly and lack cellular attachment sites [45]. By extension, the use of tyramine substitution can serve as an alternative way to control the mechanical properties of the hydrogel, other than adjusting the concentrations of HRP and H_2_O_2_ [45]. Additionally, the modulation of ingredient ratios enables precise control over the degradation rate of these composite hydrogels, with higher SF-TA or G-TA content leading to a longer lifespan [45]. Significant benefits were also observed with regard to the proliferation and thriving of human mesenchymal stem cells cultured on these composite gels, as the conjugation of cyclic arginine-glycine-aspartic acid and gelatin improved cellular attachment and morphology [45]. In vivo, implantation of these novel hydrogels under the skin of mice indicated good biocompatibility, as the inflammatory response was minimal [45].

Another novelty in biomaterials used for hydrogel fabrication is the use of an anionic exopolysaccharide (EPS) extracted from *Cryptococcus laurentii* 70766, which was functionalized with tyramine and enzymatically crosslinked using the HRP/H_2_O_2_ system [25]. The resulting hydrogel was found to be non-inferior to sodium alginate-based hydrogels while also exhibiting excellent cytocompatibility with human macrophages and fibroblast cells, with no adverse effects on their viability or proliferation [25]. These findings highlight the significant potential of *C. laurentii* 70766 EPS as a biocompatible and degradable alternative to alginate in tissue engineering applications.

### 3.8. Limitations of the Review

This review has several limitations [21]. First, it was neither systematic nor structured, which introduced the potential for selection bias. Furthermore, only two article databases were used, namely Scopus and Web of Science, suggesting that a broader database selection could yield more comprehensive results. Moreover, the search terms could be expanded to include more results, and the review did not employ the snowballing technique. Finally, the screening and analysis of articles were conducted by a limited number of reviewers.

## 4. Conclusions

The unique properties of hydrogels, along with their capacity to be fine-tuned and favorable cellular interactions, render them promising tools for TE applications in the biomedical field. The HRP/H_2_O_2_ catalytic system used for the gelation of hydrogel precursors is highly versatile, enabling precise control over the properties of the end product. Gelatin, an organic material, is both biocompatible and biodegradable, which renders it the backbone of TE applications. As presented in the current review, the applications of gelatin-based hydrogels in TE cover a broad range of tissues, with the most notable being skin, cartilage, bone, vascular, nerve, and muscle tissues. The presented HRP/H_2_O_2_ system may be used in conjunction with other crosslinking modalities in order to achieve more precise control over hydrogel properties. Moreover, a secondary polymer can be incorporated alongside gelatin to enrich the properties of the resulting hydrogel. Mathematical models can also be used to accurately predict hydrogel behavior. Modalities that prevent HRP from becoming embedded within the hydrogel should be highlighted, as they lead to a significant reduction in the immunogenic response triggered by the enzyme and therefore, enhance the suitability of HRP/H_2_O_2_ mediated hydrogels for in vivo TE applications. It should be noted that there is a lack of evidence to support the value of hydrogels for real-life human applications; thus, to improve our understanding of their utility, greater emphasis should be given to in vivo experiments with longer durations.

## Figures and Tables

**Table 1:** Extracted data from publications, including the authors, type of study, cells used, animal model, method used for hydrogel preparation/gelation, material used for the hydrogel, and application for tissue engineering.

No.	Authors	Type of Study	Cells Used	Animal Model	Method Used for Hydrogel Preparation/Gelation	Material Used for Hydrogel	Application for Tissue Engineering
1	Lu Y. et al. [22]	In vitro and in vivo	NIH-3T3 mouse embryonic fibroblasts	Sprague-Dawley (SD) rats	- Tyramine modification: Tsg and Fuc were conjugated with tyramine → Tsg-Tyr and FucC-Tyr derivatives - Enzymatic crosslinking: HRP/H_2_O_2_ system catalyzed covalent bonding between phenolic groups - Physical crosslinking: Agarose solution was cooled to form a secondary hydrogen-bonded network - Dual-network formation: Combined covalent (Tsg-Tyr/FucC-Tyr) and physical (Aga) networks	- Tilapia skin gelatin (Tsg) - Fucoidan (Fuc)- Agarose (Aga) - Tyramine (Tyr)	Skin tissue engineering (wound healing focus)
2	Nguyen T.T. [23]	In vitro	Human mesenchymal stem cells	-	- Separate HRP/H_2_O_2_ enzymatic crosslinking of tyramine-functionalized Chondroitine sulfate (CDTA) and tyramine-functionalized gelatin (GTA). - In situ formation of GTA-CDTA hybrid hydrogels by mixing solutions containing GTA, CDTA, and HRP with solutions containing GTA, CDTA, and H_2_O_2_. - Embedding BCP nanoparticles into the hybrid hydrogel by incorporating them in the polymer solution prior to enzymatic crosslinking.	- Gelatin type A from porcine skin (Bloom 300) - Chondroitine sulfate - Biphasic calcium phosphate (BCP) nanoparticles - Tyramine (TA)	Scaffold-based tissue engineering
3	Wang G. et al. [24]	In vitro and in vivo	Human vascular smooth muscle cells (VSMCs) and human aortic endothelial cells (HAECs)	Mice	- Synthesis of amine-rich, highly-branched gelatin (Gel-AE) through a nucleophilic substitution reaction. - Functionalization of Gel-AE with catechol groups through a reaction with 3,4-dihydroxyphenylacetic acid → Gel-AE-Ca precursor. - Dual crosslinking to form CAGA: covalent bonds by HRP/H_2_O_2_ crosslinking and coordinate bonds through a catechol −Fe^3+^ reaction.	- Gelatin - 2-chloroethylamine - 3,4-dihydroxyphenylacetic acid -HRP - Hydrogen peroxide (H_2_O_2_) - Iron(III) chloride (FeCl_3_)	Hemostatic bioadhesive for tissue repair
4	Hamidi M. et al. [25]	In vitro	- 3T3 L fibroblast cell line - Human macrophage - Fibroblast cell lines	-	- Functionalization of EPS and Na-Alg with tyramine hydrochloride. - HRP/H_2_O_2_ enzymatic crosslinking of the functionalized products.	- Sodium alginate (Na-Alg) - Exopolysaccharide (EPS) from *Cryptococcus laurentii* 70766 - Tyramine hydrochloride	Tissue engineering, drug delivery, and wound dressings
5	Mubarok W. et al. [26]	In vitro	- Human adipose-derived stem cells (hADS cells) - Rat fibroblast 3Y1 cells	-	- Conjugation of 3-(4-hydroxyphenyl)propionic acid with gelatin in DMF-buffered solution using WSCD-HCl → Gelatin-Ph formation. - HRP-catalyzed gelation of aqueous Gelatin-Ph solutions by supplying H_2_O_2_ from the gas phase.	- Gelatin from bovine skin (Gelatin Type B) - 3-(4-hydroxyphenyl)propionic acid - Water-soluble carbodiimide hydrochloride (WSCD-HCl)	Tissue engineering
6	Niu W. et al. [27]	In vitro and in vivo	Bone marrow mesenchymal stem cells (bMSCs)	Adult Spanish goat (for bMSC isolation)	- Gelatin (Gtn) and Hydroxyphenyl Propionic Acid (HPA) conjugation → Gtn-HPA formation. - Preparation of polyelectrolyte complex nanoparticles (PCNs) from dextran sulfate and chitosan. - Encapsulation of PDGF-BB or SDF-1α into the PCNs. - Mixing of protein-encapsulated PCNs or blank PCNs with Gtn-HPA solution, followed by HRP/ H_2_O_2_ mediated crosslinking.	-Gelatin - Hydroxyphenyl propionic acid (HPA) - Dextran sulfate (DS) - Chitosan (CS) - Platelet-derived growth factor (PDGF)-BB - Stromal cell-derived factor (SDF)-1α	Tissue engineering/regenerative medicine
7	Wang L.S. et al. [28]	In vitro and in vivo	Chondrocytes	Rabbit	- Synthesis of Gtn-HPA conjugates. - Formation of hydrogels through HRP/H_2_O_2_ mediated crosslinking. - Tunable stiffness achieved through H_2_O_2_ and Gtn-HPA concentration modulation.	- Gtn-HPA	- Cartilage tissue engineering - Osteochondral defect repair
8	Liu Y. et al. [29]	In vitro	- HAECs - Human aortic smooth muscle cells (HASMCs)	-	- Extrusion of an aqueous solution containing Alg-Ph and HRP into a flow of aqueous solution containing H_2_O_2_ and Gelatin-Ph. - Simultaneous crosslinking of Alg-Ph to form a hydrogel fiber and immobilization of Gelatin-Ph on the fiber surface via the HRP/H_2_O_2_ system.	- Alginate derivative possessing phenolic hydroxyl moieties (Alg-Ph) - Gelatin derivative possessing Ph moieties (Gelatin-Ph)	Tissue Engineering
9	Fritschen A. et al. [30]	In vitro and in vivo	- Human mesenchymal stem cells (hMSCs) - Human fibroblasts (HFF-1)	Mice	- Synthesis of Gelatin-HPA-Tyr conjugate using a two-step reaction process involving the synthesis of Gtn-HPA conjugate followed by further conjugation of Tyr. - Hydrogel formation through HRP/H_2_O_2_ oxidative coupling of phenol moieties.	- Gelatin - 3,4-hydroxyphenyl propionic acid (HPA) - Tyramine hydrochloride (Tyr.HCl) - N-hydroxysuccinimide (NHS) - 1-ethyl-3-(3-dimethylaminopropyl)-carbodiimide hydrochloride (EDC-HCI)	Tissue engineering and regenerative medicine
10	Wang L.S. et al. [31]	In vitro	hMSCs	-	- Synthesis of Gtn-HPA conjugate using carbodiimide/active ester-mediated coupling reaction. - HRP/H_2_O_2_ mediated gelation. - Tunable stiffness is achieved by varying the H_2_O_2_ concentration.	- Gtn - 3,4-Hydroxyphenylpropionic acid (HPA) - Gtn-HPA	- Neural tissue engineering - Regenerative medicine
11	Furuno K. et al. [32]	In vitro	Human embryonic kidney-derived HEK293 cells; HEK293 cells constitutively expressing GFP; HEK293 cells possessing a 35 bp deletion in the GFP sequence	-	- Electrospinning of Gelatin, containing phenolic hydroxyl moieties, produces nanofibrils. - Insolubilization of nanofibrils using horseradish peroxidase in the presence of air containing 16 ppm H_2_O_2_ for 30 min. - Loading of pDNA onto the nanofibrils through immersion in a solution of Lipofectamine/pDNA complexes.	- Gelatin from porcine skin	-Gene therapy and tissue regeneration
12	Kotani T. et al. [33]	In vitro	Mouse fibroblasts (10T1/2); Human hepatoblastoma (HepG2)	-	- For Bioprinting: Intermittent extrusion of bioink (containing cells, HRP, and phenolated polymers) and H_2_O_2_-containing support material → Improved printing fidelity. - For Scaffolds intended to culture cells: Intermittent deposition of bioink (containing a combination of HA-Ph, gelatin-Ph, and HRP, but no cells) and H_2_O_2_-containing support material → The presence of gelatin permits cell attachment and growth on the scaffolds.	- Hyaluronic acid-Ph and Gelatin-Ph	-Scaffolds and cell-laden constructs
13	Lee Y. et al. [34]	In vitro and in vivo	Human dermal fibroblasts	Nude mice; IRC mice	- In situ GH-hydrogel production (containing 7 × 10^6^ human DFBs/mL) at the exposed wound site in the presence of HRP (0.02 mg/mL) and H_2_O_2_ (0.007 wt%).	- Gelatin-hydroxyphenyl propionic acid (GH)	- Wound dressings
14	Mubarok W. et al. [35]	In vitro	C2C12 cells	-	- Addition of an aqueous solution containing 3.0% *w*/*v* Gelatin-Ph and 1 U/mL HRP in PBS to a polydimethylsiloxane (PDMS) mold (diameter: 8 mm, height: 4 mm). - Exposure to air containing H_2_O_2_ for 15, 30, 45, and 60 min follows → Hydrogels of varying consistencies.	- Gelatin from bovine	- Skeletal muscle tissue engineering
15	Zhang Y. et al. [36]	In vitro	L929 cells	-	- Gelatin and chitosan-PA + transglutaminase (TG) and HRP/H_2_O_2_ → Formation of IPN. - TG → Amide bonds between glutamine and lysine residues on adjacent gelatin chains - HRP → Crosslinking of phenol groups in chitosan-PA in the presence of H_2_O_2_.	- Gelatin and chitosan-PA	- Biocompatible scaffolds for tissue engineering and wound dressings
16	Nie K. et al. [37]	In vitro and in vivo	Human umbilical vein endothelial cells (HUVECs)	Adult male Sprague Dawley (SD) rats	- Electrospinning of Gelatin–hydroxyphenylpropionic acid (Gel–HPA) → nanofiber formation → enzymatic insolubilization through HRP/H_2_O_2_ crosslinking. - Prevention of nanofiber dissolution by an ethanol-water solution (volume ratio of 85:15).	- Gel–HPA	- Soft tissue engineering and regeneration
17	Liu X. et al. [38]	In vitro	L929 cells	-	- Gelatin and chitosan-PA + TG and HRP/H_2_O_2_ → IPN fiber formation under wet spinning conditions.	- Gelatin and chitosan-PA	- Scaffold for tissue engineering
18	Zhou F. et al. [39]	In vitro and in vivo	L929 cells	Rats; Rabbits	- Production of dopamine-modified methacrylate gelatin (GMDA) → Hydrogelation of GMDA in a two-step process; HRP/H_2_O_2_ mediated cross-linking, followed by UV light-induced photo-crosslinking (365 nm).	- GMDA	- Hemostasis, wound closure and healing
19	Moghaddam M.M. et al. [40]	In vitro	MG-63 cells	-	- Tyramine (TA) is added to hyaluronic acid (HA) and gelatin. Quantification of conjugation via UV–Vis spectroscopy and ^1^H NMR analysis. HRP/H_2_O_2_ crosslinking follows, with concentrations optimized for a 10 s gelation, ensuring spherical microglobules. - Surfactant → Rapid formation of droplets. - Sphere size = inverse to stirring speed, with the appropriate size for cell delivery being 80–100 μm.	- Hyaluronic acid (HA) and Gelatin	- Micro-scaffolds in bone tissue engineering
20	Li L. et al. [41]	In vitro and in vivo	RAW 264.7 mouse macrophages; HUVECs; Mesenchymal stem cells (MSCs)	immunocompetent C57BL/6J mice	- Covalent bonding of HRP onto porous silica particles (70–140 µm in diameter) via a polyethylene glycol molecule. - Retention of particles within the syringe during H_2_O_2_ and hydrogel precursor flow and crosslinking.	- Dextran-tyramine; Gelatin-hydroxyphenyl propionic acid	- Minimizes the immune response
21	Cheng K.W. et al. [42]	In vitro	PC12 rat pheochromocytoma cells	-	- HRA/H_2_O_2_ crosslinking of gelatin-hydroxyphenylpropionic acid. - PC12 cells embedded within the hydrogel precursor.	- Gelatin-hydroxyphenyl propionic acid	- Nerve regeneration
22	Park K.M. et al. [43]	In vitro and in vivo	HUVECs	mice	- Mixing of Gelatin–poly(ethylene glycol)–tyramine (GPT) with an aqueous solution of an angiogenic peptide in the presence of HRP/H_2_O_2_ → Hydrogel embedded with the peptide.	- GPT	- Wound healing
23	Xu S. et al. [44]	In vitro	Mouse bone marrow mesenchymal stem cells (mBMSCs)	-	- Tubular silk fibroin/gelatin-tyramine (E-SF/GT) hydrogel formation through HRP/H_2_O_2_ crosslinking and the thermosensitive properties of gelatin. - Further treatment with methanol → Distinct inner and outer layers of the EM-SF/GT tubular hydrogel.	- Silk fibroin/gelatin-tyramine	- Scaffolds for hollow multilayer tissue engineering, such as blood vessels
24	Hasturk O. et al. [45]	In vitro and in vivo	Human bone marrow mesenchymal stem cells (hMSCs)	mice	- HRP/H_2_O_2_ crosslinking of a mixture containing SF + (SF-TA or G-TA) → opaque hydrogels with quicker gelation times compared to SF alone.	- Silk fibroin (SF) - SF-TA - Gelatin-TA	- Injectable tissue fillings, 3D bioprinting or cell microencapsulation
25	Liang Y. et al. [46]	In vitro and in vivo	L929 fibroblast cells	Kunming mice, 25–30 g, female	- Chitosan + gelatin grafted with dopamine (GT-DA) + polydopamine-coated carbon nanotubes (CNT-PDA) + HRP/H_2_O_2_ → Production of hydrogels with favorable wound healing properties.	- Gelatin-grafted-dopamine (GT-DA) - Polydopamine-coated carbon nanotubes (CNT-PDA) - chitosan	- Wound dressings

## Data Availability

Data sharing is not applicable since no new data were generated.

## Abbreviations

The following abbreviations are used in this manuscript:

TE	Tissue Engineering
HRP	Horseradish Peroxidase
Tsg	Tilapia skin gelatin
Fuc	Fucoidan
SD	Sprague-Dawley
Aga	Agarose
Tyr	Tyramine
TA	Tyramine
CDTA	crosslink tyramine-functionalized Chondroitine sulfate
GTA	tyramine-functionalized gelatin
BCP	Biphasic calcium phosphate
Gel-AE	Amine-rich highly-branched gelatin
HAECs	human aortic endothelial cells
VSMCs	Human vascular smooth muscle cells
Na-Alg	Sodium alginate
EPS	Exopolysaccharide
hADS cells	Human adipose-derived stem cells
WSCD-HCl	Water-soluble carbodiimide hydrochloride
HPA	Hydroxyphenyl propionic acid
bMSCs	Bone marrow mesenchymal stem cells
Gtn-HPA	Gelatin-Hydroxyphenyl Propionic Acid
HPA	Hydroxy-phenyl pro-pionic acid
PCNs	polyelectrolyte complex nanoparticles
DS	Dextran sulfate
CS	Chitosan
PDGF	Platelet-derived growth factor
SDF	Stromal cell-derived factor
HASMCs	Human aortic smooth muscle cells
Alg-Ph	Alginate derivative possessing phenolic hydroxyl moieties
Gelatin-Ph	Gelatin derivative possessing Ph moieties
hMSCs	Human mesenchymal stem cells
Tyr.HCl	Tyramine hydrochloride
NHS	N-hydroxysuccinimide
EDC-HCI	1-ethyl-3-(3-dimethylaminopropyl)-carbodiimide hydrochloride
HepG2	Human hepatoblastoma
PDMS	polydimethylsiloxane
TG	transglutaminase
HUVECs	Human umbilical vein endothelial cells
GelMA	methacrylated gelatin
GMDA	Dopamine modified methacrylate gelatin
MSCs	Mesenchymal stem cells
GPT	Gelatin–poly(ethylene glycol)–tyramine
mBMSCs	Mouse bone marrow mesenchymal stem cells
E-SF/GT	enzymatically cross-linked silk fibroin/gelatin-tyramine
GT-DA	gelatin grafted with dopamine
CNT-PDA	polydopamine-coated carbon nanotubes
GH	Gelatin-hydroxyphenylpropionic acid
IPN	interpenetrating polymer network
chitosan-PA	chitosan containing phloretic acid
PDMS	polydimethylsiloxane
GT	gelatin–tyramine
SF	silk fibroin
gRNA	guide RNA
G-TA	gelatin-TA
EPS	exopolysaccharide
